# Alpinumisoflavone Disrupts Endoplasmic Reticulum and Mitochondria Leading to Apoptosis in Human Ovarian Cancer

**DOI:** 10.3390/pharmaceutics14030564

**Published:** 2022-03-04

**Authors:** Taeyeon Hong, Jiyeon Ham, Gwonhwa Song, Whasun Lim

**Affiliations:** 1 Department of Biological Sciences, Sungkyunkwan University, Suwon 16419, Korea; taeyeon97@g.skku.edu; 2 Institute of Animal Molecular Biotechnology and Department of Biotechnology, College of Life Sciences and Biotechnology, Korea University, Seoul 02841, Korea; glorijy76@korea.ac.kr

**Keywords:** alpinumisoflavone, ovarian cancer, apoptosis, mitochondria respiration, ER stress

## Abstract

Alpinumisoflavone is a prenylated isoflavonoid derived from the *Cudrania tricuspidate* fruit and *Genista pichisermolliana*. Alpinumisoflavone has anticancer properties in a variety of cancer cells, including colorectal, esophageal, renal and hepatocellular carcinoma. However, its mechanisms and effects in ovarian cancer remain unexplored. Our findings indicate that alpinumisoflavone triggers anti-proliferation in 2D- and 3D-cultured human ovarian cancer (ES2 and OV90) cells, including a reduction in the proliferating cell nuclear antigen expression and sub-G1 phase arrest of the cell cycle. Both alpinumisoflavone-treated ES2 and OV90 cells exhibited an augmentation in late apoptotic cells and the depolarization of mitochondrial membrane potential (MMP). We also observed a decrease in respiratory chain activity in ovarian cancer cells, owing to lower energy output by the alpinumisoflavone. In addition, combining cisplatin (a chemotherapeutic drug used in several malignancies) with alpinumisoflavone boosted apoptosis in ES2 and OV90 cells via a reduction in cell proliferation, induction of late apoptotic cells, and depolarization of MMP. Furthermore, alpinumisoflavone also regulated the PI3K/AKT, MAPK and endoplasmic reticulum (ER) stress regulatory signaling pathways, leading to cell death in both ES2 and OV90 cells. In general, our findings verified that alpinumisoflavone inhibited ovarian cancer cell growth via mitochondrial malfunction.

## 1. Introduction

Ovarian cancer (OC) is the second most common gynecological malignancy due to its high mortality rate and frequent relapses [1]. The American Cancer Society estimates that in 2021, 21,410 women will be diagnosed with OC, and among them, 13,700 are expected to die from the disease [2]. OC can be classified into three types, depending on the patient’s age. In general, it can be categorized into epithelial cell tumors in women over 50 years of age, stromal cell tumors in women of all ages, and germ cell tumors in girls under 1 year of age or 15–19 years of age [3]. According to a recent study, among OC patients that are actually diagnosed, more than 70% of them progress to stage III or IV, which is the most severe and advanced phase of the disease, and the 5-year survival rate is known to be approximately 50% [4]. Factors that increase the incidence of OC are old age, genetic alterations, family history and endometriosis. Among these factors, mutations in tumor suppression genes breast cancer type 1 (BRCA1) and BRCA2 significantly increase the risk of OC, and these cancers can be prevented by chemoprevention and bilateral ovarian resection [5]. For treating OC patients, it is important to improve the quality of systemic treatment based on surgery and chemotherapy, to increase the survival rate in first line therapy [6]. In addition, cisplatin and carboplatin are platinum-based drugs that have been used for decades and are established as the standard treatment for ovarian cancer [7]. However, most advanced OC patients suffer from chemoresistance, relapse and the side effects of therapeutic drugs. Therefore, there is a need to identify a supporting agent that can enhance cisplatin activity.

Isoflavonoids are considered as effective sensitizers for the treatment of various cancers and chronic diseases, with less side effects than conventional therapeutic agents [8]. In addition, isoflavonoids are known as phytoestrogens, as they exhibit estrogenic or antiestrogenic activities in biological responses. For example, genistein, an isoflavone, exhibits its anticancer activities by inhibiting the growth and progression of estrogen-related OC via the regulation of multiple signal transduction pathways, such as PI3K, MAPK, NF-κB and intrinsic/extrinsic apoptosis pathways [9]. In addition, the prenylation of isoflavonoids improves various pharmacological activities. Alpinumisoflavone is a prenylated isoflavonoid, and it is commonly found in the *Cudrania tricuspidate* fruit and *Genista pichisermolliana*. It is regarded as a traditional Chinese medicinal ingredient with various beneficial pharmacological features, including antiosteoporotic, antioxidative, anti-inflammatory, antibacterial and anticancer activities [10,11]. A previous study reported that alpinumisoflavone improves radiation sensitivity in esophageal squamous cell carcinoma (ESCC) [10]. Moreover, it suppresses the proliferation of lung cancer cells by the activation of intrinsic pathways and the inactivation of NF-κB and ERK1/2 MAPK pathways [12]. However, no study has been conducted on the effects of alpinumisoflavone on OC. 

Therefore, in this study, we investigated whether alpinumisoflavone regulates cell growth and programmed cell death in human OC. We demonstrated the effects of alpinumisoflavone on ES2 and OV90 cells, focusing on (1) anti-proliferation via cell cycle arrest; (2) cell death and alteration of MMP (∆Ψm); (3) impairment of mitochondrial respiration; (4) regulation of signal transduction; and (5) synergy with cisplatin. Our results suggest that alpinumisoflavone may enhance therapeutic efficiency against the growth of OC via mitochondrial dysfunction.

## 2. Materials and Methods

### 2.1. Chemicals

Alpinumisoflavone (Cat No. CFN98440) was acquired from ChemFaces and dissolved into dimethyl sulfoxide (DMSO). Cisplatin (cis-diamminedichloroplatinum) (Cat No. P4394, Sigma-Aldrich, St. Louis, MO, USA) was purchased from Sigma-Aldrich, LY294002 (Cat No. 99015) was purchased from Cell Signaling Technology (Danvers, MA, USA), and U0126 (Cat No. BML-EI282) and SB203580 (Cat. No. BML-EI286) were purchased from Enzo Life Sciences (Farmingdale, NY, USA).

### 2.2. Cell Culture of OC

ES2 and OV90 cells (OC cells) were purchased from ATCC (Manassas, VA, USA). ES2 and OV90 cells were grown in McCoy’s medium (Cat No. SH30200, Hyclone, Carlsbad, CA, USA) with a 10% fetal bovine serum (FBS) (Cat No. SH30919.03, Hyclone) and 1% penicillin–streptomycin solution (P/S) (Cat No. SV30010, Hyclone) and cultured at 37 °C in a 5% CO_2_ incubator. CHO-K1 cells, purchased from Korea Cell Line Bank (Seoul, Korea), were grown in RPMI-1640 with HEPES medium (Cat No. SH30255.01, Hyclone), 10% FBS (Cat No. SH30071.01, Hyclone) and 1% P/S at 37 °C in a 5% CO_2_ incubator. The cells were cultured using adherent methods.

### 2.3. Cell Proliferation Assay and Viability Test

The proliferation of ES2 and OV90 cells was evaluated using the bromodeoxyuridine (BrdU) Cell Proliferation ELISA kit (Cat No. 11647229001, Roche, Basel, Switzerland). Both ES2 and OV90 cells were seeded at a density of 6 × 10^3^ cells/100 μL in a 96-well cell culture plate, and then were treated with alpinumisoflavone (0, 0.5, 1, and 2 µM) or cisplatin (4 µM) for 48 h at 37 °C in a 5% CO_2_ incubator. After 48 h, BrdU was added to the cell medium and allowed to react with the cells for 2 h at 37 °C in a 5% CO_2_ incubator. After incubation for 2 h, the cells were fixed using fixation and anti-BrdU-peroxidase-working solutions were incubated at 25 °C for 1.5 h. Subsequently, the cells were washed and the same amount of substrate solution was added for detection using an ELISA reader. In addition, the cell viability of normal ovarian cells, CHO-K1, was confirmed in response to alpinumisoflavone under the same condition using MTT solution (Cat No. 11465007001, Roche, Basel, Switzerland), by following the manufacturer’s instructions. Finally, the cells were analyzed using the BioTek Epoch Plate Reader and BioTek Gen5 software. 

### 2.4. Immunofluorescence Analysis

Proliferating cell nuclear antigen (PCNA) expression was demonstrated in ES2 and OV90 cells with or without alpinumisoflavone via immunofluorescence analysis. For fluorescence imaging, cells were seeded in confocal dishes (SPL, Daejeon, Korea) and treated with alpinumisoflavone for 48 h. After incubation, the cells were washed and fixed with methanol. Subsequently, the cells were incubated with primary PCNA antibody (Santa Cruz Biotechnology, Santa Cruz, CA, USA) for 16 h. Then, we incubated the cells with anti-mouse secondary antibodies conjugated with Alexa 488 for visualization. Next, we washed the cells with PBS and stained the nuclei with DAPI. The cell images were captured using an LSM710 confocal microscope (Carl Zeiss, Oberkochen, Germany).

### 2.5. Spheroid Culture for OC

The cells were dropped on the cover of a 100 mm culture dish plate. Alpinumisoflavone-treated and nontreated ES2 and OV90 cells were cultured for 5 days by the hanging drop method. After incubation, the morphology of the spheroids was detected with a DM3000 microscope. Spheroid quantification was performed using the ImageJ software version 1.8. 

### 2.6. Migration Assay

The migration of ES2 and OV90 cells was confirmed using culture-insert 2 well in µ-dish 35 mm (Cat No. 80206, ibidi GmbH, Munich, Germany). The distances of the cell-free gap, in both with or without alpinumisoflavone groups, were captured using a DM3000 microscope (Leica, Wetzlar, Germany) and its width was quantified using the ImageJ software.

### 2.7. Cell Cycle Assay

ES2 and OV90 cells were grown to 60% confluence in a 60 mm cell culture dish. The cells were incubated with alpinumisoflavone (0, 0.5, 1 and 2 µM) for 48 h. Subsequently, the cells were collected and washed twice using 0.1% BSA in PBS, and then fixed with 70% ethanol at 4 °C for 24 h. Next, the cells were collected by centrifugation, washed with 0.1% BSA in PBS, and resuspended using a 1× binding buffer. Furthermore, 100 µL of the cell suspension was added to a brown tube and stained with RNase A (Cat No. R6513, Sigma-Aldrich) and propidium iodide (PI) for 30 min. After incubation, the stained cells were mixed with 1× binding buffer and analyzed using an FACSCalibur flow cytometer (BD Biosciences, Franklin Lakes, NJ, USA). Relative fluorescence was detected using a PE channel.

### 2.8. JC-1 Assay

To detect change in mitochondrial membrane potential, JC-1 dye (Cat No. CS0390, Invitrogen, Carlsbad, CA, USA) was employed to stain the alpinumisoflavone- or cisplatin-treated ES2 and OV90 cells. After staining with the JC-1 dye (5 μg/mL) for 20 min, the cells were washed according to the manufacturer’s instructions. Finally, the relative changes of JC-1 aggregates to JC-1 monomers were analyzed using the FL1 and FL2 channels of the flow cytometer to detect emission wavelengths of 530 nm (green, JC-1 monomer) and 590 nm (red, JC-1 aggregate).

### 2.9. Annexin V and PI Staining

ES2 and OV90 cells treated with alpinumisoflavone were validated to calculate apoptotic cells using FITC-annexin V (5 μL for each sample) and 50 mg/mL of PI of the Annexin V Apoptosis Detection Kit (Cat No. 556547, BD Biosciences). After incubation with alpinumisoflavone for 48 h, the cells were collected and stained with both dyes for 15 min. The stained cells were analyzed using the FL1 and FL2 channels of the flow cytometer.

### 2.10. Western Blotting

ES2 and OV90 cells were treated with alpinumisoflavone (0, 0.5, 1 and 2 µM) for 24 h. Proteins were extracted using a whole cell lysate buffer. Denatured proteins were loaded in each well of 10% acrylamide gel in equal amounts (calculated via the Bradford assay) and subjected to SDS-PAGE. After separating the proteins via electrophoresis, the separated proteins in the gels were transferred onto nitrocellulose membranes. Thereafter, primary and secondary antibodies (Cat No. 5450-00110, SeraCare, MA, USA) were incubated with the membranes to detect the target proteins. The immunoblots were detected via chemiluminescence and visualized using the ChemiDoc EQ system (Bio-Rad, Hercules, CA, USA). The antibodies used in immunoblotting assays are listed in Table 1. 

### 2.11. Mitochondrial Respiration Measurement Using the Seahorse XFe24 Analyzer

For the mitochondrial stress analysis, ES2 and OV90 cells were seeded in Seahorse XFe24 cell culture microplates at a concentration of 3 × 10^4^ cells/100 μL. After the cell confluency reached 80% in each well, the cells were treated with alpinumisoflavone (2 µM) for the next 24 h; then, they were additionally treated with 1.5 µM of oligomycin (an inhibitor of ATP synthase), 0.5 µM of carbonyl cyanide 4-(trifluoromethoxy) phenylhydrazone (also known as FCCP, an uncoupler), and a mixture of 0.5 µM rotenone (a mitochondrial complex I inhibitor) and 0.5 µM antimycin A (a mitochondrial complex III inhibitor) during the measurement, according to the user guide of the Seahorse XF Cell Mito Stress Test Kit (Cat No. 103015-100, Agilent Technologies, Santa Clara, CA, USA) and previous studies [13,14]. The treated cells were evaluated for oxygen consumption rate (OCR) using a Seahorse XFe24 Analyzer (Agilent Technologies).

### 2.12. Statistical Analysis

To verify that ES2 and OV90 cells exhibited significant differential effects in response to treatments, all triplicate data results were subjected to analysis of variance by least-squares analysis of variance (ANOVA), according to the general linear model (PROC-GLM) of the SAS program (SAS Institute, Cary, NC, USA). Differences with a probability value of * *p* < 0.05 were considered statistically significant. The obtained data are presented as the mean ± SEM, unless otherwise stated.

## 3. Results

### 3.1. Alpinumisoflavone Regulated the Proliferation of OC Cells

**Figure 1:**
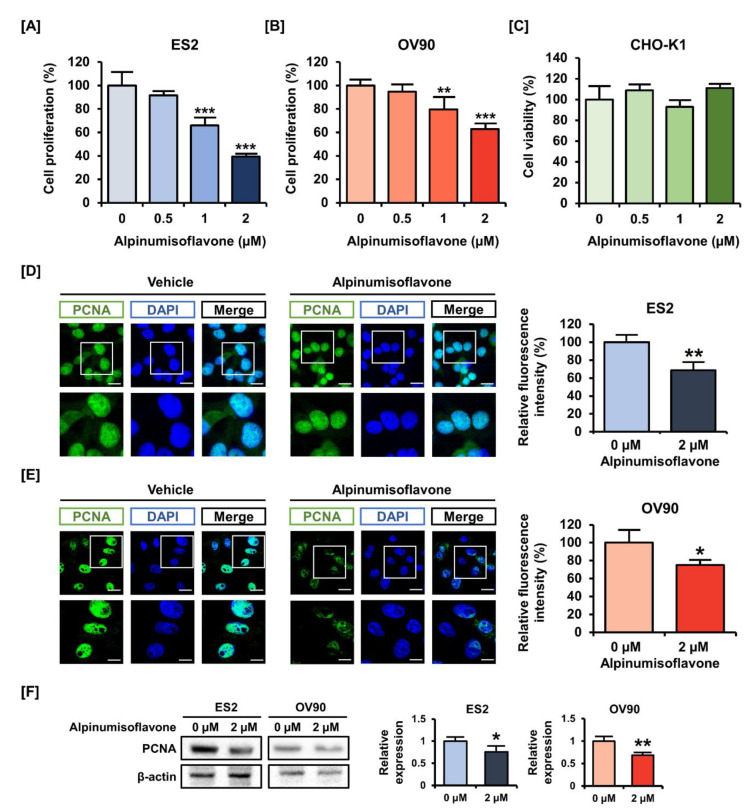
Effects of alpinumisoflavone on the proliferation of human ovarian cancer cells. (**A**,**B**) Cell proliferation assay was adopted to detect the proliferation of ES2 and OV90 cells in response to alpinumisoflavone (0, 0.5, 1 and 2 μM) for 48 h. The results were compared to cells that had been treated with a vehicle. (**C**) Cell viability of CHO-K1, normal ovarian cells, was analyzed. (**D**,**E**) PCNA proteins are depicted by green fluorescence, while DAPI counterstaining for nuclei is represented by blue fluorescence. Cells were treated with alpinumisoflavone for 48 h. Metamorph software was employed to measure the relative intensity of the green fluorescence compared to vehicle-treated cells. The scale bar displayed 20 μm (the first horizontal line) and 40 μm (the second horizontal line). (**F**) Immunoblots of PCNA in response to alpinumisoflavone (2 μM) treatment in ES2 and OV90 cells. All experiments were performed in triplicate. According to the adopted standards, the level of significance between the vehicle and treatment groups is depicted by asterisks (* *p* < 0.05, ** *p* < 0.01 and *** *p* < 0.001).

Epithelial OC cells (ES2 and OV90) were exposed to alpinumisoflavones (0, 0.5, 1 and 2 µM) for 48 h to estimate cell proliferation. In response to the dose-dependent treatment of alpinumisoflavone, the cell proliferations of ES2 and OV90 gradually decreased (Figure 1A,B). Specifically, the cell proliferations decreased to 39% (*p* < 0.001) and 69% (*p* < 0.001) in ES2 and OV90 cells with 2 µM alpinumisoflavone, respectively. In addition, we exposed CHO-K1 cells to alpinumisoflavone under the same conditions as for ES2 and OV90, to check the toxicity of alpinumisoflavone in normal ovarian cells (Figure 1C). The obtained results indicated that alpinumisoflavone did not alter the cell viability of CHO-K1 cells. Based on the reduced proliferation of OC cells by alpinumisoflavone, we compared the immunofluorescence intensity of PCNA in ES2 and OV90 cells, with or without alpinumisoflavone (2 µM). The treatment of alpinumisoflavone significantly decreased the immunoreactive PCNA expression in both ES2 and OV90 cells (Figure 1D,E). We further confirmed the suppression of PCNA expression in response to alpinumisoflavone treatment in both cell lines via western blot analysis (Figure 1F).

### 3.2. Alpinumisoflavone Inhibited Cell Growth and the Migration of ES2 and OV90 Cells

**Figure 2:**
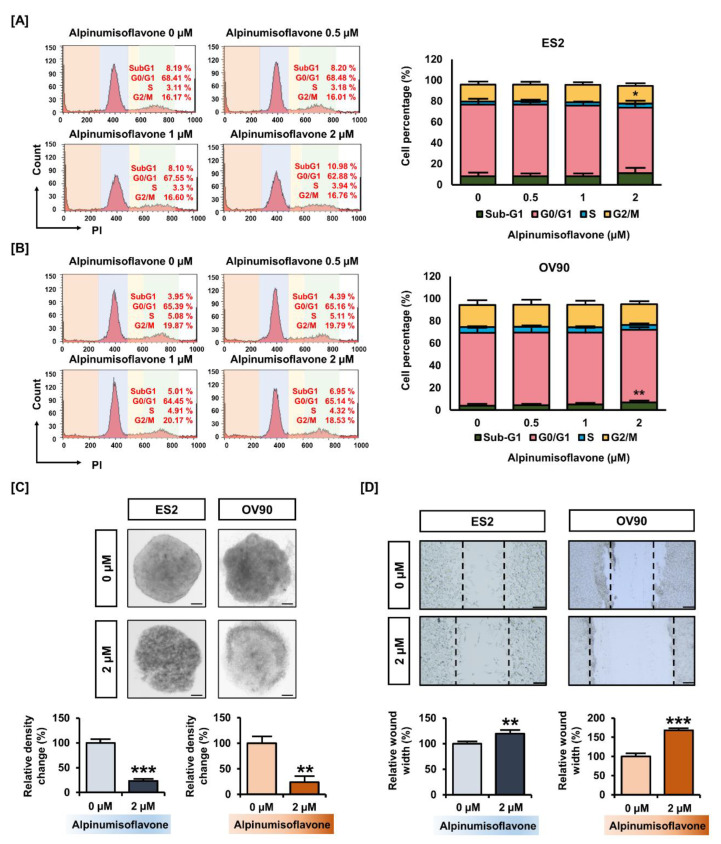
Change in ovarian cancer cell growth triggered by alpinumisoflavone. (**A**,**B**) Histogram illustration of cell cycle progression in alpinumisoflavone-treated (0, 0.5, 1 and 2 M) ovarian cancer cells for 48 h. The percentages of cells in the sub-G1, G0/G1, S and G2/M phases are presented in the comparative graph. (**C**) 3D-spheroid formation in ovarian cancer cells as examined by hanging-drop cell culture methods for cells treated with vehicle and alpinumisoflavone for 5 days. (**D**) Gap distance was calculated for detecting the migration of ES2 and OV90 cells in response to alpinumisoflavone. All experiments were performed in triplicate. The level of significance between the vehicle and treatment groups was depicted by asterisks (* *p* < 0.05, ** *p* < 0.01 and *** *p* < 0.001).

Next, we determined whether alpinumisoflavone induces cell cycle arrest in OC cells. The proportion of ES2 and OV90 cells treated with 2 µM alpinumisoflavone in the sub-G1 phase increased by 2.89% and 3.0%, respectively, but the change was only significant in OV90 cells (Figure 2A,B). In addition, the proportion of cells in the S-phase of the ES2 population significantly increased (Figure 2A). To verify the anti-proliferation effect, we analyzed the spheroid formation of ES2 and OV90 cells in an alpinumisoflavone-included medium. The obtained results indicate that the 3D spheroid density of ES2 and OV90 cells decreased by approximately 80% (*p* < 0.001), compared to vehicle-treated spheroid cells (Figure 2C). In addition, ES2 and OV90 cells were cultured using culture-insert 2 well in µ-dish to measure cancer cell migration. The alpinumisoflavone treatment inhibited cell migration by increasing the gap distance between cells by 1.2- and 1.7-fold in ES2 and OV90 cell cultures, respectively, compared to vehicle-treated cells (Figure 2D). Collectively, alpinumisoflavone suppressed cell growth in ES2 and OV90 cells.

### 3.3. Alpinumisoflavone Induced Cell Death and the Depolarization of MMP (∆Ψm) in ES2 and OV90 Cells

**Figure 3:**
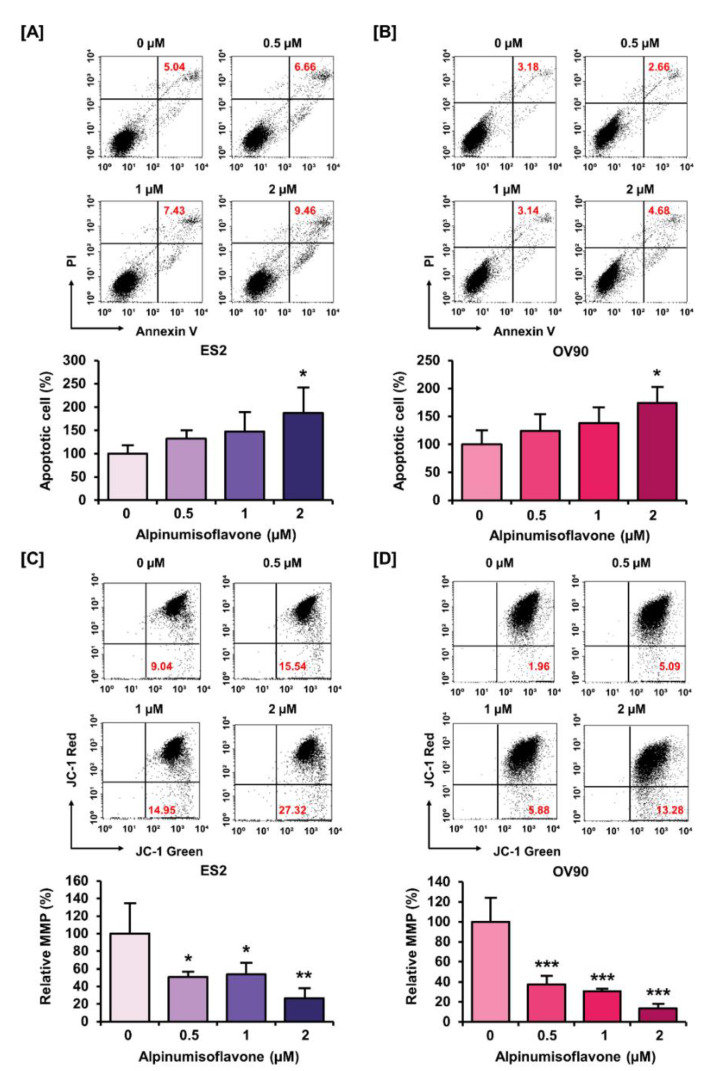
Alteration of mitotic membrane potential (MMP) and late apoptotic cell number by alpinumisoflavone in both cell lines. (**A**,**B**) The death of ES2 and OV90 cells treated with alpinumisoflavone for 48 h was determined using annexin V and PI assay. (**C**,**D**) The effect of alpinumisoflavone on the disruption of MMP was investigated using JC-1 assay. Relative JC-1 red/green population is represented as a bar graph in percentage-ratio under the flow cytometry data. All experiments were performed in triplicate. The level of significance between the vehicle and treatment groups was depicted by asterisks (* *p* < 0.05, ** *p* < 0.01 and *** *p* < 0.001).

We investigated the population of late apoptosis cells using an annexin V staining kit, as well as the change in MMP using JC-1 dye in ES2 and OV90 cells with alpinumisoflavone. First, annexin V staining results indicated that alpinumisoflavone (2 μM) increased the amount of late apoptotic OV90 and ES2 cells by 187% (*p* < 0.05) and 165% (*p* < 0.05), respectively, compared to the vehicle-treated group (Figure 3A,B). Furthermore, MMP was disrupted by 77% (*p* < 0.01) and 87% (*p* < 0.001) in ES2 and OV90 cells, respectively, in response to alpinumisoflavone treatment (Figure 3C,D). Together, the results revealed that alpinumisoflavone triggered cell death with the depolarization of MMP in ES2 and OV90 cells.

### 3.4. Alpinumisoflavone Regulated Mitochondrial Respiration in OC Cells

**Figure 4:**
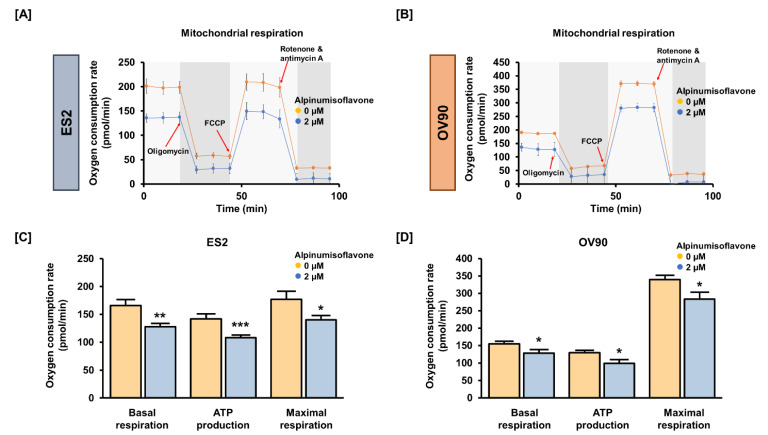
In ES2 and OV90 cells, alpinumisoflavone regulates mitochondrial energy metabolism. (**A**,**B**) The Seahorse Mito Stress test was adopted to examine the OCR of ES2 and OV90 cells after alpinumisoflavone (2 μM) treatment. Oligomycin (1.5 μM), FCCP (0.5 μM) and rotenone/antimycin A (0.5 μM) were serially administered. (**C**,**D**) Bar graphs indicate data from the Seahorse analysis for basal respiration, ATP production and maximal respiration. The control ES2 and OV90 cells are represented by orange-colored lines or bar graphs, while the alpinumisoflavone-treated ES2 and OV90 cells are represented by blue-colored lines or bar graphs. The level of significance between the vehicle and treatment groups is depicted by asterisks (* *p* < 0.05, ** *p* < 0.01 and *** *p* < 0.001).

To detect OCR, we conducted Seahorse XF Cell Mito Stress tests using the Seahorse XFe analyzer. We verified mitochondrial respiration in OC cells using oligomycin (1.5 μM), FCCP (0.5 μM) and rotenone/antimycin A (0.5 μM). Basal and maximal respiration were significantly reduced by approximately 20% in both cell lines following treatment with 2 μM alpinumisoflavone. In addition, alpinumisoflavone significantly reduced ATP generation by approximately 25% in ES2 and OV90 cells compared to control cells (Figure 4). Therefore, alpinumisoflavone inhibited mitochondrial respiration, followed by mitochondrial malfunction.

### 3.5. Alpinumisoflavone Showed a Synergistic Effect with Cisplatin in Suppressing the Proliferation of OC Cells

**Figure 5:**
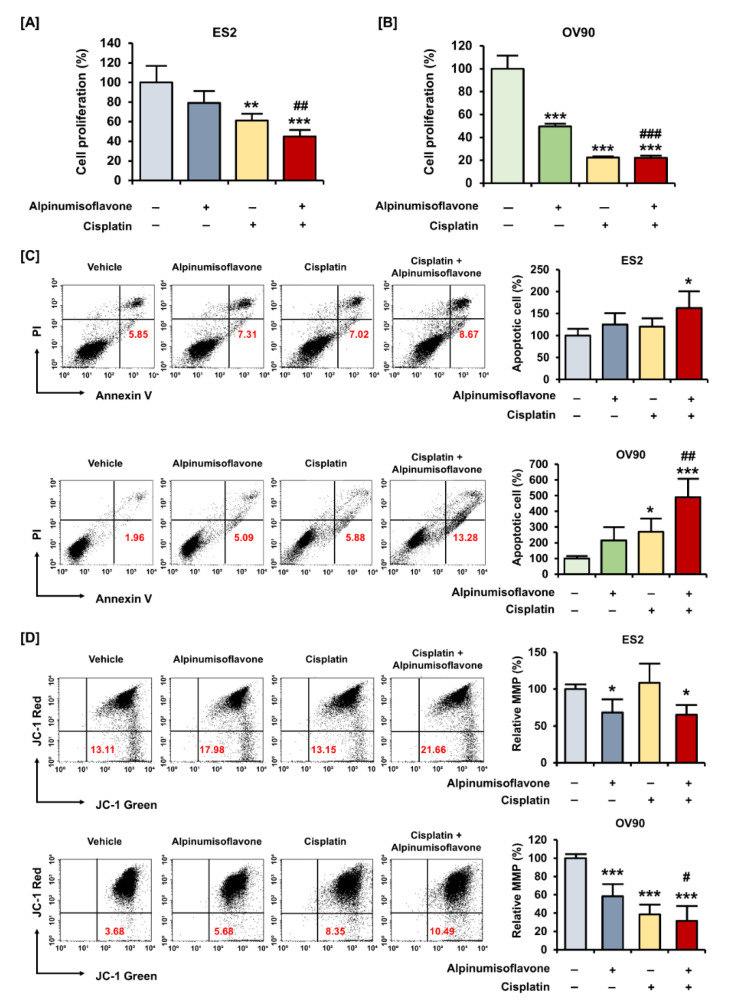
Alpinumisoflavone with cisplatin exhibited synergistic effects on suppression of proliferation of ovarian cancer cells. (**A**,**B**) A cell proliferation assay was used to detect the proliferation of ES2 and OV90 cells in response to cisplatin, with or without alpinumisoflavone treatment, for 48 h. (**C**) Apoptosis of ES2 and OV90 cells in response to cisplatin (4 μM), with or without alpinumisoflavone (2 μM), was investigated using annexin V and PI. Relative late apoptotic cells are presented as bar graphs compared to the vehicle-treated cells (100%). (**D**) A JC-1 assay was conducted to study the effect of alpinumisoflavone, or a combination of alpinumisoflavone with cisplatin, on MMP. The MMP condition is indicated by the quadrant of the dot blot using flow cytometry. All experiments were performed in triplicate. The level of significance between the vehicle and treatment groups is depicted by asterisks (* *p* < 0.05, ** *p* < 0.01 and *** *p* < 0.001). The level of significance between alpinumisoflavone and the co-treatment groups is depicted by crosshatch (^#^ *p* < 0.05, ^##^ *p* < 0.01 and ^###^ *p* < 0.001).

To measure cell proliferation, human OC (ES2 and OV90) cells were treated with alpinumisoflavone, or a combination of alpinumisoflavone with cisplatin, for 48 h. The results indicated that ES2 and OV90 cells treated with alpinumisoflavone (2 μM) and cisplatin (4 μM) exhibited a more significant decrease in proliferation compared to cells treated with alpinumisoflavone alone (Figure 5A,B). To verify the synergistic effect of alpinumisoflavone with cisplatin on mitochondrial-regulated cell death, we determined the death of apoptotic cells and the depolarization of MMP in the OC cells (Figure 5C,D). The proportion of apoptotic ES2 cells was significantly increased after cisplatin treatment with alpinumisoflavone (Figure 5C). In addition, OV90 cells treated with alpinumisoflavone significantly increased upon the additional treatment with cisplatin, compared to those treated with alpinumisoflavone alone. Furthermore, the permeability of MMP in OV90 cells was significantly disrupted by a combination of alpinumisoflavone and cisplatin; however, there was no significant effect on ES2 cells (Figure 5D). These results indicate that the combined treatment exhibited complementary effects on the inhibition of cellular proliferation, thereby leading to the death of OC cells.

### 3.6. Alpinumisoflavone Regulated Signaling Proteins Related to Proliferation and Endoplasmic Reticulum (ER) Stress in ES2 and OV90 Cells

**Figure 6:**
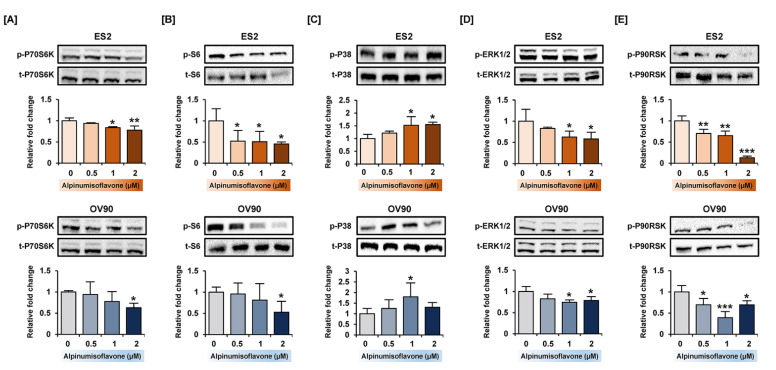
Relative intensity of proteins belonging to PI3K and MAPK signaling pathway in ES2 and OV90 cells treated with alpinumisoflavone. (**A**–**E**) Phosphorylation of P70S6K (**A**), S6 (**B**), P38 (**C**), ERK1/2 (**D**) and P90RSK (**E**) proteins in immunoblots as analyzed in ES2 and OV90 cells at different alpinumisoflavone doses (0, 0.5, 1 and 2 µM) for 24 h. The standardized values of the phosphorylated proteins are normalized by the intensity of total protein levels. All experiments were performed in triplicate. The significance between the vehicle and treatment groups are depicted by asterisks (* *p* < 0.05, ** *p* < 0.01 and *** *p* < 0.001).

**Figure 7:**
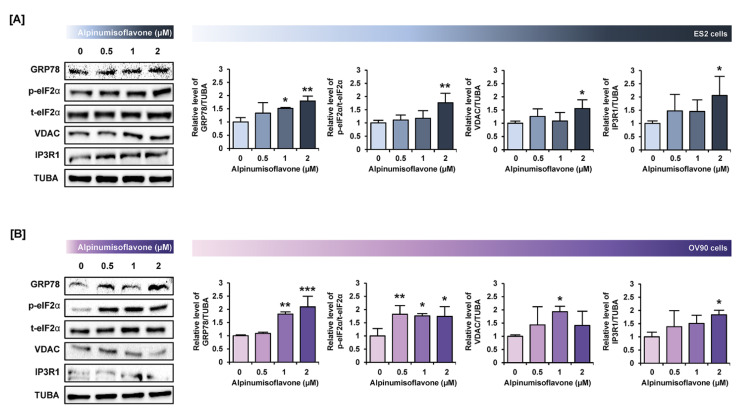
Alpinumisoflavone activates the ER stress and ER–mitochondria contact proteins in ES2 and OV90 cells. (**A**,**B**) In both ES2 (**A**) and OV90 (**B**) cells treated with alpinumisoflavone for 24 h, immunoblots of GRP78, phosphor-eIF2α, total-eIF2α, VDAC, IP3R1 and TUBA were captured using the ChemiDoc system. Relative intensities of each target protein are presented as bar graphs next to sorted immunoblots. All experiments were performed in triplicate. The significance between the vehicle and treatment groups is depicted by asterisks (* *p* < 0.05, ** *p* < 0.01 and *** *p* < 0.001).

To verify the change in protein levels triggered by alpinumisoflavone, we conducted a western blot analysis. Downstream of the PI3K signaling pathway, the phosphorylation of P70S6K and S6 proteins was gradually reduced in both cell lines in response to alpinumisoflavone (Figure 6A,B). Furthermore, the expression of phosphor-P38 protein in alpinumisoflavone-treated cell lines increased, compared to vehicle-treated cells (Figure 6C). In addition, the phosphor-ERK1/2 and P90RSK proteins slightly decreased with alpinumisoflavone in both ES2 and OV90 cells (Figure 6D,E). Subsequently, we investigated the relative levels of unfolded protein in ES2 and OV90 cells treated with different doses of alpinumisoflavone. The phosphor-eIF2α and GRP78 proteins were promoted by alpinumisoflavone in both cells (Figure 7). In addition, the expression levels of VDAC and IP3R1 proteins, which are involved in the ER–mitochondria axis for regulating calcium ion levels, were upregulated in response to alpinumisoflavone in both ES2 and OV90 cells. In general, alpinumisoflavone altered the levels of signaling molecules regulating proliferation, ER stress and ER-mitochondrial contact in the OC cells.

### 3.7. Alpinumisoflavone with Inhibitors of Target Pathways Induced Cell Apoptosis and Inhibited Intracellular Signaling

**Figure 8:**
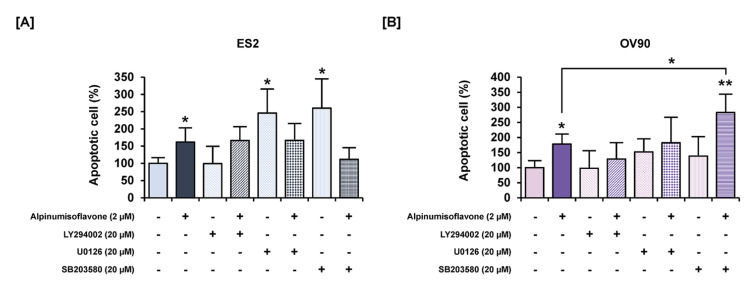
Effects of a combination of each inhibitor for PI3K, ERK1/2 and P38 MAPK pathways, with or without alpinumisoflavone, on apoptosis in ovarian cancer cells. (**A**,**B**) Apoptosis of ES2 (**A**) and OV90 (**B**) cells in response to LY294002, U0126 and SB203580, with or without alpinumisoflavone treatment for 48 h, was investigated using annexin V and PI. All experiments were performed in triplicate. The significance between the vehicle and treatment groups is depicted by asterisks (* *p* < 0.05 and ** *p* < 0.01).

**Figure 9:**
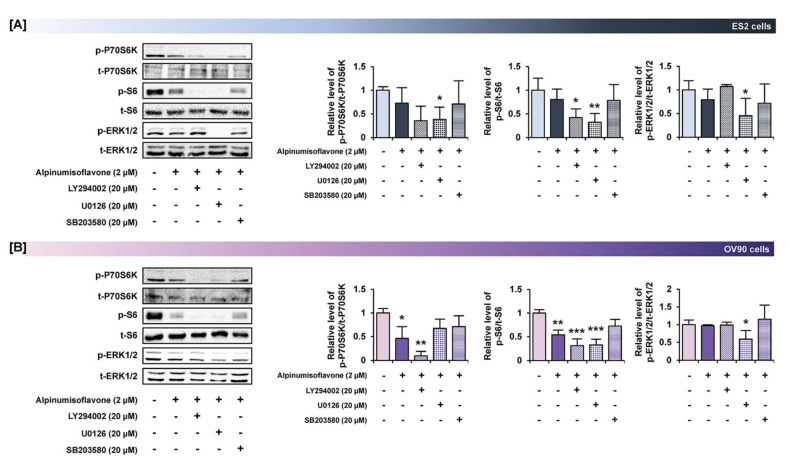
Effects of pretreatment of inhibitors for PI3K, ERK1/2 and P38 MAPK pathways prior to alpinumisoflavone treatment in ovarian cancer cells. (**A**,**B**) Western blot analysis revealed alterations in the phosphorylation of target proteins after pretreatment with each inhibitor for 1 h prior to alpinumisoflavone treatment. In ES2 and OV90 cells, a comparative graph represents the change in phosphorylation relative to the vehicle-treated control (100%). All experiments were performed in triplicate. The significance between the vehicle and treatment groups is depicted by asterisks (* *p* < 0.05, ** *p* < 0.01 and *** *p* < 0.001).

To determine the synergistic effect of alpinumisoflavone with pharmacological inhibitors, we performed annexin V/PI staining and western blot analyses. The amount of late apoptosis cells increased following the pretreatment with LY294002 (PI3K inhibitor), U0126 (ERK1/2 inhibitor) and SB203580 (P38 inhibitor) prior to the treatment of alpinumisoflavone, compared to the sole treatment of each inhibitor in OV90 cells, whereas there were no significant differences between a single treatment of target substance and the combined treatment (Figure 8). Furthermore, in both ES2 and OV90 cells, the phosphorylation of P70S6K was better suppressed by all these inhibitors than with alpinumisoflavone alone (Figure 9). The phosphorylation of S6 was blocked by the inhibitors, except SB203580, in both cell lines. The phosphorylation of ERK1/2 was blocked by a combination of alpinumisoflavone with U0126 in ES2 and OV90 cells. In general, alpinumisoflavone regulated the OC cell proliferation via PI3K and MAPK signaling.

## 4. Discussion

**Figure 10:**
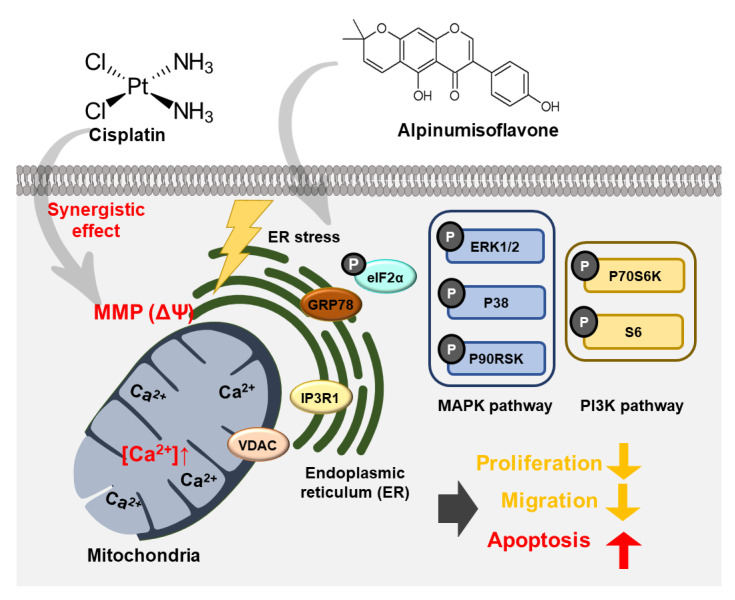
Schematic illustration of possible molecular mechanisms induced by alpinumisoflavone treatment in ES2 and OV90 cells.

In this study, alpinumisoflavone suppressed cell proliferation, PCNA expression, spheroid formation and cell migration in human OC cells (Figure 10). Furthermore, alpinumisoflavone promoted the loss of MMP and increased the number of late apoptotic cells. In addition, cisplatin, a chemotherapeutic agent administered against cancers, exhibited similar effects in combination with alpinumisoflavone, thus enhancing the efficiency of alpinumisoflavone in treating OC cells. Moreover, alpinumisoflavone triggered mitochondrial dysfunction and anti-proliferative effects in OC cells through the regulation of MAPK, PI3K and ER stress signaling pathways. Collectively, we elucidated the anticancer effects and cellular mechanisms of alpinumisoflavone in human OC cells.

Platinum-based anticancer drugs are marketed worldwide, and with approximately one-half of patients being treated with cisplatin, cisplatin is one of the best metal-based chemotherapy drugs [15]. Although cisplatin has been proven to be effective against a variety of cancers, including sarcoma, cancers of the bone, muscle, etc., the drug’s resistance and significant side effects have inspired a novel treatment strategy for the management of various cancers with the combination of cisplatin and other drugs or supplementation [16]. In previous studies, natural compounds, such as fucoidan and eupatilin, were adopted to improve chemotherapeutic effects with additional chemotherapy by inhibiting proliferation and angiogenesis [17,18]. In this study, cisplatin increased the mitochondria-regulated cell death in ES2 and OV90 cells when the cells were treated with alpinumisoflavone, compared to the case where cisplatin was used alone.

Mitochondrial dysfunction, which is caused by mtDNA mutations or mitochondrial enzyme deficiencies, disrupts cellular bioenergetics and supports cancer cell metabolic reprogramming; in addition, it triggers tumor-promoting alterations mediated by reactive oxygen species, Ca^2+^, or small molecule metabolites released from mitochondria [19]. Furthermore, mitochondria serve as the cell's powerhouse, producing over 80% of ATP and performing several cellular functions. Mitochondria maintain cell motility and intracellular homeostasis and are closely related to apoptosis [20]. For example, α,β-thujone disrupt mitochondrial function via metabolic alterations by reducing maximal respiration, ATP production and MMP, leading to cell death in ovarian cancer [21]. In particular, inhibiting mitochondrial respiration in ovarian cancer could enhance the sensitivity to anticancer drugs even in chemo-resistant cells, because of its high dependence on the OXPHOS system [22]. Although alpinumisoflavone has a role in inhibiting the proliferation of cancerous cells, the effects of alpinumisoflavone remain unclear. In this study, we identified that alpinumisoflavone induced the disruption of mitochondrial respiration and the depolarization of MMP, which results in the cell death of ES2 and OV90 cells.

The PI3K and MAPK signaling system is a significant therapeutic target for OC, and the inhibition of this mechanism can impede cancer growth [23]. In a previous study, it was reported that the management of the PI3K pathway is a critical strategy for treating OC patients using specific inhibitors [24]. In addition, the inhibition of PI3K and ERK1/2 pathways exhibited synergistic effects on inhibiting tumor growth in OC [25]. Similar to previous studies, alpinumisoflavone inhibited the PI3K and ERK1/2 MAPK pathways in OC cells, leading to apoptosis, while pharmacological inhibitors, including LY294002 or U0126, in combination with alpinumisoflavone, improved the antiproliferative effects of alpinumisoflavone on the OC cells. 

ER is an organelle involved in multiple processes, including protein homeostasis, stress response and Ca^2+^ homeostasis [26]. GRP78 aids in the stabilization of ER proteins and triggers the unfolded protein response. In addition, surface-associated GRP78 is involved in cytoprotection and mediated cytoskeletal remodeling, while secreted GRP78 is involved in immunomodulation [27]. High levels of GRP78 can increase the activities of matrix metalloproteinases in pancreatic cancer metastasis and invasion by activating signaling pathways, such as JNK and FAK [28]. In addition, ER stress leading to excessive GRP78 and eIF2α expression was determined to be closely related to the activation of the P38 protein in tumor cells [29,30]. VDACs located in the outer mitochondrial membrane are responsible for the release of cytochrome C from the mitochondrial membrane space into the cytoplasm, which is an early step in the apoptosis process [31]. IP3R1, located in the endoplasmic reticulum, is known to play important roles, such as cell proliferation, metabolism and apoptosis, by pumping calcium from intracellular calcium stores. In addition, IP3R1 overexpression induces an increase in cytoplasmic Ca^2+^ levels and apoptosis [32]. Likewise, the IP3R-mediated increase in calcium ion flux between the ER and mitochondria sensitized cisplatin sensitivity and activated ER stress in OC [33]. In OC cells, alpinumisoflavone activated ER stress sensor proteins and ER–mitochondria axis proteins with the activation of P38 protein, thus indicating that alpinumisoflavone has anticancer effects mediated by the ER–mitochondria axis. 

## 5. Conclusions

In summary, we first demonstrated the anticancer effects of alpinumisoflavone on OC cells, and further explained the working mechanisms of alpinumisoflavone in OC cells. Therefore, we suggest alpinumisoflavone as a potential therapeutic agent against OC cells. Considering its synergistic effect with cisplatin, it can be used as a supplementary agent to treat chemoresistance. However, since our studies were limited to cell lines, further in vivo and clinical verification and validation will be needed for future applications.

## Figures and Tables

**Table 1:** Antibodies used in immunoblotting assays.

Antibodies	Catalog No.	Supplier	Dilution
PCNA	sc-56	Santa Cruz	1:1000
β-actin	sc-47778	Santa Cruz	1:1000
p-P70S6K (Thr^421^/Ser^424^)	9204	Cell Signaling Technology (CST)	1:1000
p-S6 (Ser^235^/Ser^236^)	2211	CST	1:1000
p-P38 (Thr^180^/Tyr^182^)	4511	CST	1:1000
p-ERK1/2 (Thr^202^/Tyr^204^)	9101	CST	1:1000
p-P90RSK (Ser^573^)	9346	CST	1:1000
t-P70S6K	9202	CST	1:1000
t-S6	2217	CST	1:1000
t-P38	9212	CST	1:1000
t-ERK1/2	4695	CST	1:1000
RSK1/RSK2/RSK3	9355	CST	1:1000
p-eIF2α (Ser^122^)	3398	CST	1:1000
GRP78	sc-13968	Santa Cruz	1:1000
IP3R1	PA1-901	Invitrogen	1:1000
VDAC	4661	CST	1:1000
t-eIF2α	5324	CST	1:1000
TUBA	sc-32293	Santa Cruz	1:2000

## Data Availability

Data are contained within the article.

## References

[1] Bae H., Song G., Lim W. (2020). Stigmasterol Causes Ovarian Cancer Cell Apoptosis by Inducing Endoplasmic Reticulum and Mitochondrial Dysfunction. Pharmaceutics.

[2] De Simone F.I. The Need for Early Detection in Ovarian Cancer. https://www.mlo-online.com/disease/cancer/article/21222611/the-need-for-early-detection-in-ovarian-cancer.

[3] Roett M.A., Evans P. (2009). Ovarian cancer: An overview. Am. Fam. Physician.

[4] Stewart C., Ralyea C., Lockwood S. (2019). Ovarian Cancer: An Integrated Review. Semin. Oncol. Nurs..

[5] Daly M.B., Pilarski R., Berry M., Buys S.S., Farmer M., Friedman S., Garber J.E., Kauff N.D., Khan S., Klein C. (2017). NCCN Guidelines Insights: Genetic/Familial High-Risk Assessment: Breast and Ovarian, Version 2.2017. J. Natl. Compr. Cancer Netw..

[6] Ledermann J.A. (2018). First-line treatment of ovarian cancer: Questions and controversies to address. Ther. Adv. Med. Oncol..

[7] Dilruba S., Grondana A., Schiedel A.C., Ueno N.T., Bartholomeusz C., Cinatl J., McLaughlin K.M., Wass M.N., Michaelis M., Kalayda G.V. (2020). Non-Phosphorylatable PEA-15 Sensitises SKOV-3 Ovarian Cancer Cells to Cisplatin. Cells.

[8] Cayetano-Salazar L., Olea-Flores M., Zuniga-Eulogio M.D., Weinstein-Oppenheimer C., Fernandez-Tilapa G., Mendoza-Catalan M.A., Zacapala-Gomez A.E., Ortiz-Ortiz J., Ortuno-Pineda C., Navarro-Tito N. (2021). Natural isoflavonoids in invasive cancer therapy: From bench to bedside. Phytother. Res..

[9] Lee J.Y., Kim H.S., Song Y.S. (2012). Genistein as a Potential Anticancer Agent against Ovarian Cancer. J. Tradit. Complement. Med..

[10] Zhang B., Fan X., Wang Z., Zhu W., Li J. (2017). Alpinumisoflavone radiosensitizes esophageal squamous cell carcinoma through inducing apoptosis and cell cycle arrest. Biomed. Pharmacother..

[11] Ateba S.B., Mvondo M.A., Djiogue S., Zingue S., Krenn L., Njamen D. (2019). A Pharmacological Overview of Alpinumisoflavone, a Natural Prenylated Isoflavonoid. Front. Pharmacol..

[12] Namkoong S., Kim T.J., Jang I.S., Kang K.W., Oh W.K., Park J. (2011). Alpinumisoflavone induces apoptosis and suppresses extracellular signal-regulated kinases/mitogen activated protein kinase and nuclear factor-kappaB pathways in lung tumor cells. Biol. Pharm. Bull..

[13] Al-Masri M., Paliotti K., Tran R., Halaoui R., Lelarge V., Chatterjee S., Wang L.T., Moraes C., McCaffrey L. (2021). Architectural control of metabolic plasticity in epithelial cancer cells. Commun. Biol..

[14] Romani C., Capoferri D., Grillo E., Silvestri M., Corsini M., Zanotti L., Todeschini P., Ravaggi A., Bignotti E., Odicino F. (2021). The Claudin-Low Subtype of High-Grade Serous Ovarian Carcinoma Exhibits Stem Cell Features. Cancers.

[15] Ghosh S. (2019). Cisplatin: The first metal based anticancer drug. Bioorg. Chem..

[16] Dasari S., Tchounwou P.B. (2014). Cisplatin in cancer therapy: Molecular mechanisms of action. Eur. J. Pharmacol..

[17] Bae H., Lee J.Y., Yang C., Song G., Lim W. (2020). Fucoidan Derived from Fucus vesiculosus Inhibits the Development of Human Ovarian Cancer via the Disturbance of Calcium Homeostasis, Endoplasmic Reticulum Stress, and Angiogenesis. Mar. Drugs.

[18] Lee J.Y., Bae H., Yang C., Park S., Youn B.S., Kim H.S., Song G., Lim W. (2020). Eupatilin Promotes Cell Death by Calcium Influx through ER-Mitochondria Axis with SERPINB11 Inhibition in Epithelial Ovarian Cancer. Cancers.

[19] Srinivasan S., Guha M., Kashina A., Avadhani N.G. (2017). Mitochondrial dysfunction and mitochondrial dynamics-The cancer connection. Biochim. Biophys. Acta Bioenerg..

[20] Singh K.K. (2004). Mitochondrial dysfunction is a common phenotype in aging and cancer. Ann. N. Y. Acad. Sci..

[21] Lee J.Y., Park H., Lim W., Song G. (2021). Therapeutic potential of alpha, beta-thujone through metabolic reprogramming and caspase-dependent apoptosis in ovarian cancer cells. J. Cell. Physiol..

[22] Emmings E., Mullany S., Chang Z., Landen C.N., Linder S., Bazzaro M. (2019). Targeting Mitochondria for Treatment of Chemoresistant Ovarian Cancer. Int. J. Mol. Sci..

[23] Liu Z., Zhang S., Hou F., Zhang C., Gao J., Wang K. (2019). Inhibition of Ca(2+)-activated chloride channel ANO1 suppresses ovarian cancer through inactivating PI3K/Akt signaling. Int. J. Cancer.

[24] Li H., Zeng J., Shen K. (2014). PI3K/AKT/mTOR signaling pathway as a therapeutic target for ovarian cancer. Arch. Gynecol. Obstet..

[25] Sheppard K.E., Cullinane C., Hannan K.M., Wall M., Chan J., Barber F., Foo J., Cameron D., Neilsen A., Ng P. (2013). Synergistic inhibition of ovarian cancer cell growth by combining selective PI3K/mTOR and RAS/ERK pathway inhibitors. Eur. J. Cancer.

[26] Bhat T.A., Chaudhary A.K., Kumar S., O’Malley J., Inigo J.R., Kumar R., Yadav N., Chandra D. (2017). Endoplasmic reticulum-mediated unfolded protein response and mitochondrial apoptosis in cancer. Biochim. Biophys. Acta Rev. Cancer.

[27] Lu G., Luo H., Zhu X. (2020). Targeting the GRP78 Pathway for Cancer Therapy. Front. Med..

[28] Malhi H., Kaufman R.J. (2011). Endoplasmic reticulum stress in liver disease. J. Hepatol..

[29] Darling N.J., Cook S.J. (2014). The role of MAPK signalling pathways in the response to endoplasmic reticulum stress. Biochim. Biophys. Acta.

[30] Park G.B., Kim Y.S., Lee H.K., Song H., Cho D.H., Lee W.J., Hur D.Y. (2010). Endoplasmic reticulum stress-mediated apoptosis of EBV-transformed B cells by cross-linking of CD70 is dependent upon generation of reactive oxygen species and activation of p38 MAPK and JNK pathway. J. Immunol..

[31] Hoogenboom B.W., Suda K., Engel A., Fotiadis D. (2007). The supramolecular assemblies of voltage-dependent anion channels in the native membrane. J. Mol. Biol..

[32] Tsunoda T., Koga H., Yokomizo A., Tatsugami K., Eto M., Inokuchi J., Hirata A., Masuda K., Okumura K., Naito S. (2005). Inositol 1,4,5-trisphosphate (IP3) receptor type1 (IP3R1) modulates the acquisition of cisplatin resistance in bladder cancer cell lines. Oncogene.

[33] Xie Q., Xu Y., Gao W., Zhang Y., Su J., Liu Y., Guo Y., Dou M., Hu K., Sun L. (2018). TATfused IP3Rderived peptide enhances cisplatin sensitivity of ovarian cancer cells by increasing ER Ca^2+^ release. Int. J. Mol. Med..

